# Simultaneous Whole-Chamber Non-contact Mapping of Highest Dominant Frequency Sites During Persistent Atrial Fibrillation: A Prospective Ablation Study

**DOI:** 10.3389/fphys.2022.826449

**Published:** 2022-03-16

**Authors:** Gavin S. Chu, Xin Li, Peter J. Stafford, Frederique J. Vanheusden, João L. Salinet, Tiago P. Almeida, Nawshin Dastagir, Alastair J. Sandilands, Paulus Kirchhof, Fernando S. Schlindwein, G. André Ng

**Affiliations:** ^1^Department of Cardiovascular Science, University of Leicester, Leicester, United Kingdom; ^2^Lancashire Cardiac Centre, Blackpool Teaching Hospitals NHS Foundation Trust, Blackpool, United Kingdom; ^3^School of Engineering, University of Leicester, Leicester, United Kingdom; ^4^National Institute for Health Research Leicester Cardiovascular Biomedical Research Centre, Glenfield Hospital, Leicester, United Kingdom; ^5^School of Science and Technology, Nottingham Trent University, Nottingham, United Kingdom; ^6^Center for Engineering, Modeling and Applied Social Sciences, University Federal of ABC, Santo André, Brazil; ^7^Department of International Foundation, Massey University, Auckland, New Zealand; ^8^University Heart and Vascular Centre, University Medical Center Hamburg-Eppendorf, Hamburg, Germany

**Keywords:** atrial fibrillation, catheter ablation, non-contact mapping, atrial electrograms, dominant frequency, persistent AF, multi-layer, rotors

## Abstract

**Purpose:**

Sites of highest dominant frequency (HDF) are implicated by many proposed mechanisms underlying persistent atrial fibrillation (persAF). We hypothesized that prospectively identifying and ablating dynamic left atrial HDF sites would favorably impact the electrophysiological substrate of persAF. We aim to assess the feasibility of prospectively identifying HDF sites by global simultaneous left atrial mapping.

**Methods:**

PersAF patients with no prior ablation history underwent global simultaneous left atrial non-contact mapping. 30 s of electrograms recorded during AF were exported into a bespoke MATLAB interface to identify HDF regions, which were then targeted for ablation, prior to pulmonary vein isolation. Following ablation of each region, change in AF cycle length (AFCL) was documented (≥ 10 ms considered significant). Baseline isopotential maps of ablated regions were retrospectively analyzed looking for rotors and focal activation or extinction events.

**Results:**

A total of 51 HDF regions were identified and ablated in 10 patients (median DF 5.8Hz, range 4.4–7.1Hz). An increase in AFCL of was seen in 20 of the 51 regions (39%), including AF termination in 4 patients. 5 out of 10 patients (including the 4 patients where AF termination occurred with HDF-guided ablation) were free from AF recurrence at 1 year. The proportion of HDF occurrences in an ablated region was not associated with change in AFCL (τ = 0.11, *p* = 0.24). Regions where AFCL decreased by 10 ms or more (i.e., AF disorganization) after ablation also showed lowest baseline spectral organization (*p* < 0.033 for any comparison). Considering all ablated regions, the average proportion of HDF events which were also HRI events was 8.0 ± 13%. Focal activations predominated (537/1253 events) in the ablated regions on isopotential maps, were modestly associated with the proportion of HDF occurrences represented by the ablated region (Kendall’s τ = 0.40, *p* < 0.0001), and very strongly associated with focal extinction events (τ = 0.79, *p* < 0.0001). Rotors were rare (4/1253 events).

**Conclusion:**

Targeting dynamic HDF sites is feasible and can be efficacious, but lacks specificity in identifying relevant human persAF substrate. Spectral organization may have an adjunctive role in preventing unnecessary substrate ablation. Dynamic HDF sites are not associated with observable rotational activity on isopotential mapping, but epi-endocardial breakthroughs could be contributory.

## Introduction

Atrial fibrillation (AF) is the commonest cardiac arrhythmia in clinical practice, affecting 2% of the population worldwide (Nattel, 2002). AF increases the risk of stroke fivefold and is associated with increased mortality (Nattel, 2002). Catheter ablation is an effective therapy for paroxysmal AF (pAF) (Haissaguerre et al., 1998; Fichtner et al., 2015), but the identification of successful ablation targets in patients with persistent AF (persAF) remains challenging (Jalife et al., 2002; Nattel, 2002, 2003). The electrophysiological mechanisms underlying persAF and current adjunctive ablation strategies beyond pulmonary vein isolation (PVI) lack clear evidence for effectiveness (Providencia et al., 2015; Verma et al., 2015; Mohanty et al., 2018). Recently, endocardial-epicardial interaction has been highlighted as a relevant pathophysiological contributor (Yamazaki et al., 2012; Gutbrod et al., 2015; Hansen et al., 2015), but this has not yet been translated into the clinical arena.

Sheep optical mapping studies (Mandapati et al., 2000; Mansour et al., 2001; Kalifa et al., 2006) first outlined the potential of using dominant frequency (DF) assessment to detect AF driver sites, predicated around the observation of rotors (Mandapati et al., 2000), but the utility of DF is also implicit with other proposed mechanisms (Kumagai et al., 2000; Lin et al., 2005; Kalifa et al., 2006). DF has previously demonstrated good correlation with local cycle length (Earley et al., 2006; Lin et al., 2007; Gojraty et al., 2009). Despite this, human ablation studies based on point-by-point sequential DF mapping were inconclusive (Atienza et al., 2009, 2014; Verma et al., 2011). Highest DF (HDF) sites have since been shown to be spatiotemporally unstable (Lazar et al., 2004; Yokoyama et al., 2009; Habel et al., 2010; Jarman et al., 2012; Salinet et al., 2014); consequently, as a natural corollary, simultaneous multisite mapping is necessary to reliably localize atrial high DF areas.

In this study, we hypothesized that the strategy of prospectively identifying and ablating dynamic left atrial HDF sites would favorably impact the electrophysiological substrate of persAF. We sought in particular to assess the feasibility of prospectively identifying HDF sites by global simultaneous left atrial mapping across long continuous time segments, and to describe the underlying wavefront activation characteristics at these sites.

## Materials and Methods

### Patients

Ten persAF patients with no previous ablation history gave written informed consent to undergo HDF mapping and ablation, on uninterrupted oral anticoagulation. All had undergone successful direct current cardioversion (DCCV) previously, and median AF duration (from the first documented AF post-DCCV up to the time of their procedure) was 219 (range 132–848) days. Table 1 summarizes the clinical characteristics of the group. The study was independently approved by the United Kingdom national health research ethics service. Procedures were performed under general anesthesia. All anti-arrhythmic drugs (AADs) were stopped for at least 5 half-lives, except amiodarone which was continued. Every patient was in AF at the start of their procedure.

**TABLE 1:** Clinical and procedural characteristics of patients with and AF recurrence within 12 months following ablation.

	All patients	AF free at 12 months	AF-recurrence within 12 months
*N*	10	5	5
Age/years	57.7 ± 12.1	57.3 ± 9.0	58.2 ± 14.6
Body mass index/kg m^–2^	31.0 ± 5.7	32.6 ± 6.7	29.5 ± 3.7
Longstanding persistent AF	3	2	1
LA volume/ml	151 ± 38	146 ± 40	156 ± 35
Amiodarone usage	2	2	0
Hypertension	3	1	2
Diabetes mellitus	1	0	1
Previous myocardial infarction	1	1	0
Procedure duration/mins	389 ± 80	386 ± 65	393 ± 92
LA area ablated during HDF targeting/mm^2^ (% of LA total)	1362 ± 704 (7.3 ± 3.6)	1055 ± 494 (5.9 ± 3.1)	1670 ± 746 (8.7 ± 3.6)
HDF occurrences ablated (%LA)	559 ± 268 (22.8 ± 8.7)	448 ± 278 (21.6 ± 7.1)	670 ± 205 (23.9 ± 9.9)
Electrical cardioversion required at procedure end to restore sinus rhythm	5	0	5

*Numbers are mean ± SD where relevant. HDF, highest dominant frequency; LA, left atrium.*

### Non-contact Mapping

A non-contact multi-electrode array (Ensite Array, St. Jude Medical, St Paul, MN, United States) was positioned transseptally in the left atrium (LA) alongside an EZ Steer Thermocool ablation catheter (Biosense Webster, Diamond Bar, CA, United States). Patients were heparinized to maintain an activated clotting time > 300 s. 3D electroanatomic mapping was performed using the Velocity platform (St. Jude Medical). 30 s of continuous AF activity were recorded, and the virtual electrograms (vEGMs) of a 2048 node geometry from this period were exported.

### Signal Processing

**FIGURE 1:**
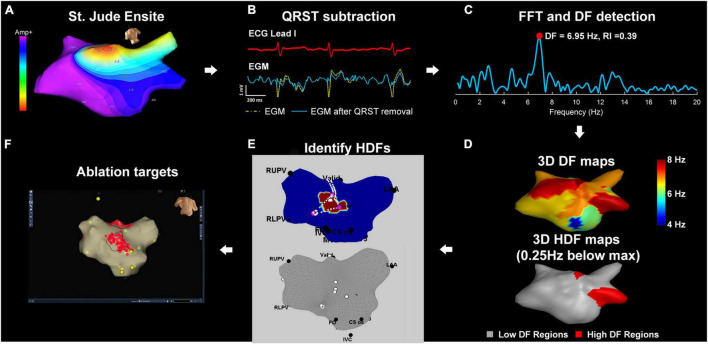
Diagram of the workflow for ablation targets identification. **(A)** St. Jude Ensite: left atrial geometry isopotential map exported from Ensite Velocity System. **(B)** Array data is imported into a bespoke MATLAB user interface. QRST subtraction: Electrograms using one ECG lead as reference. **(C)** FFT and DF detection: power spectrum of the current non-contact atrial signal and DF identification **(D)** 3D and 2D DF/HDF maps: MATLAB reconstructed 3D Atrial geometry with color-coded DF/HDF and transformation to 2D uniform grid. **(E)** The top panel shows an antero-posterior view of the LA, with the region hosting HDF for a single 4 s time window highlighted in purple. The pink dots indicate the HDF spatial centers for all time windows. For better intraprocedural clarity, the bottom panel shows only the HDF spatial centers (white dots) identified across all the mapped time windows. **(F)** Identifying and ablating HDF regions. These are transcribed into the Velocity 3D mapping system and targeted with ablation (red dots). Yellow dots represent anatomical marker points. FFT, fast Fourier transform; HDF, highest dominant frequency.

A bespoke MATLAB graphical user interface was created for the study (Li et al., 2017), incorporating our previously published spectral analysis methodology (Salinet et al., 2014; Li et al., 2021), generating 13 sequential DF maps in each patient with 30 s data. The non-contact MEA catheter was used to collect intracardiac signals, as previously described. 2,048 channels of virtual electrograms (vEGMs) were sampled at 2034.5 Hz and exported with a 1–150 Hz filter setting from Ensite system (Figure 1A). MATLAB was used to analyze the data offline (Mathworks, United States). As shown in Figure 1B, ventricular far-field activity was removed from the recorded vEGMs using a previously described QRST subtraction technique (Salinet et al., 2013). The vEGMs were then divided into 4 s window segments that overlapped by 50%. The fast Fourier transform (FFT) was used to perform spectral analysis on each segment (Figure 1C). A Hamming window was applied to the atrial vEGMs to reduce leakage. To improve DF identification, zero padding was used, resulting in a frequency step of 0.05 Hz. The peak in the power spectrum within the physiological range of 4–10 Hz was defined as DF (Figure 1C) (Salinet et al., 2014). Regularity index (RI) was defined as the ratio of spectral area (power) under the curve centered at DF peak (0.75 Hz bandwidth) and area under the full physiological spectrum (here 4 – 20 Hz, Figure 1C) (Sanders et al., 2005).

### Highest Dominant Frequency Ablation Targeting

For each 4 s window, HDF occurrences were defined as all nodes hosting a DF within 0.25 Hz of the maximum DF for that map (shown as purple on the LA geometry in the example in the top panel of Figure 1D). To avoid biasing for target size, the spatial centers of the HDF occurrence regions for each map were projected onto the LA geometry in MATLAB (bottom panel, Figure 1E). The intended regions of ablation were transcribed on to the Velocity geometry, with the objective of prospectively defining several discrete regions for ablation. Each region where possible would encompass multiple co-localizing HDF spatial centers which would be ablated “en-bloc” (Figure 1F). Once this initial map was created, changes or re-mapping were not permitted.

### Ablation Protocol

Highest dominant frequency spatial centers were targeted for radiofrequency ablation, with the objective of eliminating local atrial signal. The bipolar signal at the LAA is invariably well demarcated and permits unambiguous manual assessment of AFCL, has been applied as a surrogate of AF organization in many other clinical studies (O’Neill et al., 2006, 2009; Haissaguerre et al., 2007; Rostock et al., 2011; Honarbakhsh et al., 2018a). Following each region of HDF-guided ablation, AFCL in the left atrial appendage (LAA) was measured using the ablation catheter over 10 cycles to evaluate ablation response. A 10 ms change in AFCL was considered *a priori* to be significant (Bezerra et al., 2020). This was repeated until one of the following pre-defined endpoints was reached:

1) Termination of AF to sinus rhythm (SR);
2) Conversion from AF to an organized LA rhythm, or;
3) Operator decision to stop based on satisfactory target coverage or patient safety.

A further post-procedural Velocity data export was performed to capture all radiofrequency (RF) point (lesion) locations corresponding to each ablation region. Every RF point has an associated location on the LA geometry (the closest atrial endocardial surface point). Regularity index (RI) was defined as the ratio of spectral area (power) under the curve of DF peak and area under the full spectrum. Therefore, each point was associated with a DF value and an RI value which both vary over time. The DF and RI values at these LA geometry points were averaged spatially and temporally to generate (scalar) mean DF and RI values for each ablated region individually. There was no attempt to manually filter ablation points.

Finally, the Array was removed and replaced by PVAC (Pulmonary Vein Ablation Catheter, Medtronic, Fridley, MN, United States) to achieve PVI, irrespective of the atrial rhythm. Where necessary, intravenous flecainide followed by DCCV was delivered to restore SR at the end of the procedure.

### Associating Post-ablation AF Cycle Length Change With Regional Pre-ablation Spectral Characteristics

Each of the 51 ablated regions across the whole patient cohort was categorized by the AFCL change arising from ablation in the region. The DIS group was pre-defined as regions where ablation resulted in a reduction in AFCL (i.e., DISorganization) by 10 ms or more. The ORG group was pre-defined as regions where ablation resulted in AFCL increase by 10 ms or more, or termination of arrhythmia (i.e., ORGanization). All other regions were classified as EQUivocal (i.e., an AFCL change of 9 ms or less in either direction).

Highest dominant frequency was defined as above, while highest RI (HRI) was defined as the top decile of RI values for the LA within any single given time window. HDF + HRI concurrence was defined whenever a given LA geometry point hosted HDF and HRI in the same time window.

HDF, HRI, and HDF + HRI concurrence was retrospectively compared across the DIS, EQU, and ORG groups.

### Isopotential Map Wavefront Analysis

**FIGURE 2:**
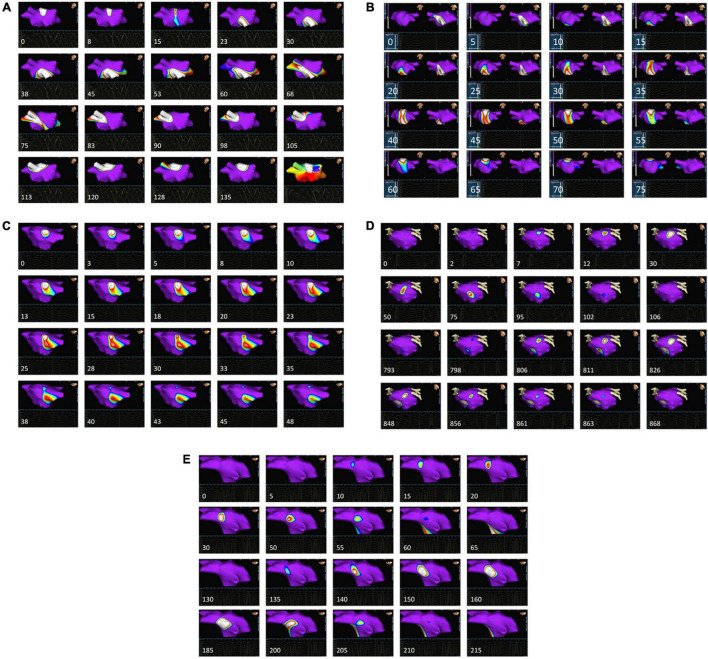
Patterns of pre-ablation isopotential map behavior in and around HDF regions. For each case, the temporal sequence is from left to right and top to bottom. The timing of each frame relative to the first is given in ms. Each image is centered around an area that was subsequently ablated based upon the presence of HDF spatial centers. Purple areas on the map represent atrial myocardium where local activation is absent, as defined by a local vEGM (virtual electrogram) amplitude above –0.28 mV. Voltages of –0.53 mV or less display as white, with the remainder of the color scale defining intermediate values. The appearance and trajectory of color around the maps were used to define the following wavefront activation patterns: **(A)** Rotor-like behavior, seen on the LA roof. The final panel shows an isochronal map of the area during this period, confirming rotational activity. AFCL was not significant altered by ablation in this region. (see also Supplementary Video 1). **(B)** Activation passes through the posterior wall of the LA three times consecutively (see Supplementary Video 2) within a single TQ period, but ablation here did not alter AFCL. **(C)** A wavefront is seen to split into two independent wavefronts on the LA roof, with the division occurring within the ablated area. AFCL was not significantly affected by ablation here (see also Supplementary Video 3). **(D)** Focal activation occurs near the left upper pulmonary vein, migrating out of the ablated area before extinguishing, as also demonstrated in Supplementary Video 4A. Later on, the same area is seen to activate again from an identical origin (Supplementary Video 4B), this time extinguishing within the lesion. Ablation here terminated AF to an atrial tachycardia. **(E)** A recurring focal extinction event, occurring on the LA roof. Focal activation arises outside the ablated region, then moves into and extinguishes within the ablated area (first 10 images). This behavior is repeated again shortly afterward (last 10 images) within the same TQ interval. See also Supplementary Video 5.

A retrospective analysis of the pre-ablation patterns of activation behavior in HDF regions was performed in the Velocity environment using the following pre-specified protocol. Each discrete region that received ablation was circumscribed on the geometry. The isopotential mapping area was then centered upon this region. Activation was defined when local vEGM voltage fell below the fixed thresholds of either −0.28 or −0.53 mV (Hoshiyama et al., 2016). The rationale for these thresholds is based on the work of Hoshiyama and colleagues, where endocardial mapping of the LA was performed using the same non-contact multielectrode array as the one in the present study (Hoshiyama et al., 2016). In their study, vEGM signals from premature atrial contractions (PACs) were recorded at the time of spontaneous onset of AF. In particular, very short-coupled PACs (VS-PACs) were defined as “PAC with the shortest coupling interval that was observed just before the AF onset.” The amplitude of the vEGM during VS-PACs was reported as 0.53 ± 0.25 mV. This threshold represented the smallest amplitude for a PAC that would have been associated with discrete ECG evidence of relevant activation, and was therefore used to define the lower activation threshold of −0.53 mV and the upper threshold of −0.28 mV (one standard deviation above the lower threshold) as used in the present study. The described approach avoided reliance upon more arbitrary amplitude thresholds during AF, with such thresholds inevitably being smaller and hence unfavorably reducing overall signal-noise ratio. Playback of the isopotential map from the 30 s period corresponding to the time of HDF mapping was performed, looking to document specific pre-defined activation trajectories encompassing current mechanistic theories of AF persistence (see Figure 2 for detailed examples, and also the video links available in Supplementary Materials). Examples of the considered behaviors are provided in Figure 2, and Supplementary Video links are available in Supplementary Materials. Events were pre-defined as specific visually observed behaviors of activation encompassing current mechanistic theories of AF:

1) Rotor (Narayan et al., 2012, 2014) – core must remain in the lesion with a circular activation path of at least 360 degrees.
2) Critical pathway involved in single or multiple loop re-entry (Lin et al., 2005) – entry and exit of >50% of the activation wavefront must be from distinct sides of the lesion.
3) Wavelet propagation (Moe and Abildskov, 1959; Moe et al., 1964; Allessie, 1985) – Division of a primary wavefront into two or more separate wavefronts occurring within the lesion.
4) Focal wavefront activation (Haissaguerre et al., 1998; Kumagai et al., 2000) – wavefront spontaneously emerges radially from within an otherwise non-activated lesion.
5) Focal wavefront extinction (de Groot et al., 2010, 2016) – wavefront enters from outside the lesion, reduces radially and extinguishes within the lesion.

For each ablated region, the frequency of each of the above behaviors within the 30 s segment was counted (see Supplementary Videos for examples). The observer was blinded to the AFCL change. Events partially or entirely within the QRST period were ignored.

The consistency of focal activation events was evaluated within each ablation region individually by assessing the maximum and minimum number of focal events over the prior 10 TQ intervals, creating a “moving maximum” (MMax) and “moving minimum” (MMin). The difference between the greatest and least value of MMin and MMax over the 30 s period was designated “diffMMin” and “diffMMax,” respectively.

### Clinical Follow-Up

Following a 3-month blanking period, patients underwent at least 24 h of continuous ambulatory ECG monitoring, and recurrence was defined as any documented AF of at least 30 s occurring between 3 and 12 months post-procedure, irrespective of ongoing AADs.

### Statistical Analysis

Data normality was assessed visually and using the Kolmogorov-Smirnov test. Correlations were performed using Spearman’s or Kendall’s method depending on the presence of rank ties, within MATLAB or using Prism v7.03 (Graphpad Software, CA, United States). Pairwise comparisons between groups were performed using the “TPB20” percentile bootstrap method with 20% trimmed means (Wilcox, 2012). Non-parametric trends analyses were performed using the Jonckheere-Terpstra test. Statistical significance was defined at the 0.05 level, and further adjusted for multiple comparisons. Both linear and logistic mixed effects regression models were explored but did not add utility (*p* = 1.00 and *p* = 0.27, respectively) for non-zero between-patient variance in AFCL outcome, (R v3.2.1, R Foundation for Statistical Computing, Vienna, Austria).

## Results

### Clinical Outcomes

All patients completed the study protocol. Procedure duration was 390 ± 57 min, in keeping with a novel mapping and ablation protocol. RF time ablating HDF regions was 54 ± 27 min, covering an LA ablation area of 1447 ± 676 mm^2^, corresponding to 7.8 ± 3.6% of the total mapped LA area, prior to PVI.

**FIGURE 3:**
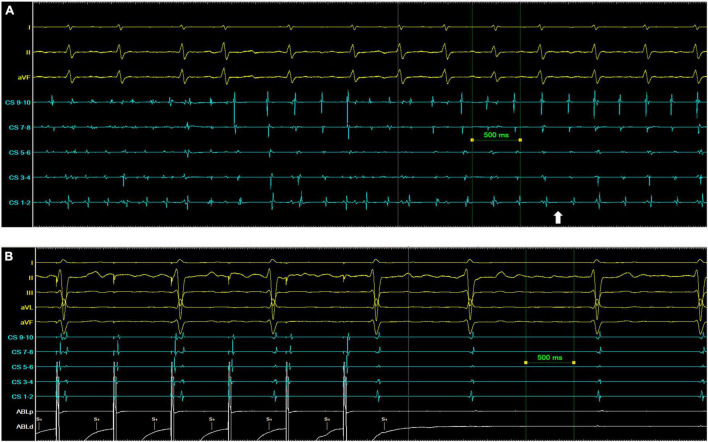
Examples of AF termination following ablation of a region of highest dominant frequency. **(A)** Patient 5. The white arrow indicates the point of transition from AF to a persistent organized atrial tachyarrhythmia. **(B)** Patient 7. The left atrium is silent with no coronary sinus (CS) signal at baseline, but with ECG evidence of ongoing AF. Pacing from the ablation (Abl) catheter captures the CS with organized distal to proximal activation.

**FIGURE 4:**
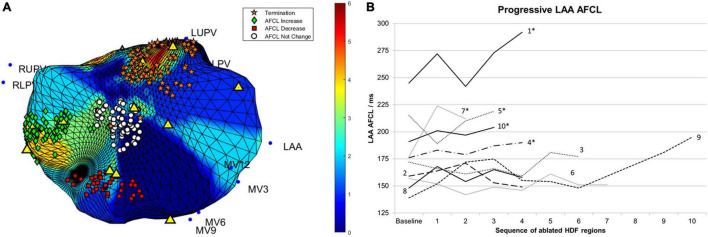
**(A)** The four HDF targeted ablation regions from patient 5 are shown. The color scale corresponds to occurrences of HDF at the given spatial location. Individual lesions are labeled according to their impact on AFCL, with a change of 10 ms or more considered significant. Yellow triangles indicate the location of HDF spatial centers. **(B)** Changes in AFCL for each consecutive region of HDF-guided LA ablation. Lines are labeled with their respective patient number. Case progression is from left to right. *, patients in whom sinus rhythm was restored without the need for electrical cardioversion; AFCL, atrial fibrillation cycle length; HDF, highest dominant frequency; MV, mitral valve annular locations; LUPV, left upper pulmonary vein; RUPV, right upper pulmonary vein; RLPV, right lower pulmonary vein.

Five patients converted to SR without the need for DCCV. Patient 1 (longstanding persAF, on amiodarone) converted with flecainide after PVI. Patients 10 (longstanding persAF, on amiodarone), 5 (Figure 3A) and 4 converted from AF to atrial flutter, and patient 7 converted transiently to LA silence (Figure 3B) before then terminating to SR (all with HDF-guided ablation alone, prior to PVI). AF termination sites were the base of LAA, the LA roof (in 2 patients), and the posterior wall. An example of the ablation performed is shown in Figure 4A.

No significant adverse events occurred. During the 12-month follow-up period, all 5 patients requiring DCCV at the end of their procedure experienced AF recurrence, in contrast to zero out of the 5 who ended their procedure in SR without the need for DCCV. Table 1 lists the clinical characteristics of patients with and without recurrent AF.

### Characteristics of 30 s Highest Dominant Frequency-Guided Ablation Regions and AF Cycle Length Responses

The pre-ablation global LA mean DF was strongly correlated with baseline AFCL (r = 0.88, *p* < 0.001). A total of 51 discrete regions were ablated during the study, 20 (39%) of which resulted in significant AFCL increase or termination, as summarized in Table 2. Ablated region size was 267 ± 290 mm^2^. The averaged DF for each ablated region was 5.7 ± 0.7 Hz with an average RI of 0.35 ± 0.06. A median of 4 (range 3–10) regions of ablation were delivered per patient.

**TABLE 2:** Location of ablated regions targeted using HDF mapping, and their associated left atrial response.

	Termination	AFCL increase	AFCL unchanged	AFCL decrease
Anterior	0	2	2	1
Posterior	1	3	5	3
Roof	2	3	9	0
Septum	0	4	0	1
Left PV region	0	2	3	1
Right PV region	0	0	4	2
Left atrial appendage	1	1	0	0
Lateral	0	1	0	0

*AFCL, atrial fibrillation cycle length; PV, pulmonary vein.*

Figure 4B shows the AFCL response to prospectively targeted ablation of consecutive HDF regions, demonstrating: (1) higher baseline AFCL conferred greater likelihood of achieving SR without DCCV (*p* < 0.01); (2) HDF-targeted ablation could disorganize as well as organize AF, but; (3) this did not preclude subsequent AF organization and/or termination. Only one patient had a further significant increment in AFCL following PVI (Patient 9, from 195 to 222 ms).

Median lesion size was 166 (21–1380) mm^2^. The area of ablation alone (debulking) was not associated with AFCL variation (Kendall’s τ = 0.05, *p* = 0.64).

The proportion of HDF occurrences per ablated region (compared with the entire LA across 30 s) ranged from 0 to 14.7% (median 2.6%). Correlation between this and AFCL change was non-significant (τ = 0.11, *p* = 0.24).

### Highest Dominant Frequency and Highest RI Occurrences in Ablated Regions

**FIGURE 5:**
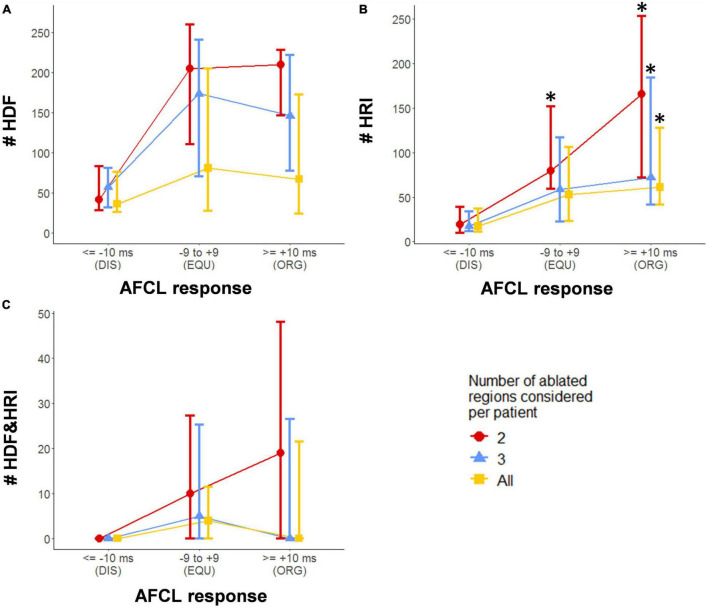
Spectral characteristics of HDF-targeted ablation regions compared with the AFCL response to ablation of that region. **(A)** HDF counts. **(B)** HRI counts. **(C)** HDF + HRI concurrence counts. Values shown are median ± interquartile range. DIS – ablation lesions resulting in an AFCL decrease/disorganization of 10 ms or more; EQU – ablation lesions resulting in equivocal change in AFCL of between –9 and + 9 ms; ORG – ablation lesions resulting in AFCL increase/organization of 10 ms or more; * indicates a statistically significant difference when compared with the corresponding DIS group after correction for multiple comparisons.

The relationship between the spectral behavior of ablated regions and the AFCL response to ablation was assessed by comparing the number of HDF and HRI occurrences between the AFCL response groups, as shown in Figure 5. In view of the prolonged RF delivery times and varying extent of ablation, the possibility of cumulative ablation effects was assessed by evaluating the above metrics for only the first two (indicated in red) and first three (indicated in green) ablated regions for each patient, and finally for all ablated regions (indicated in blue).

Highest RI showed statistically significant trends analyses, as well as differences between the DIS group and the ORG group, for all extents of ablation. A significant difference was also seen in HRI between the DIS and EQU group when considering only the first two lesions. No other trends or comparisons were statistically relevant. Considering all ablated regions, the average proportion of HDF events which were also HRI events was 8.0 ± 13%.

For each patient in this study, DF mapping utilized a total of 13 consecutive time windows of 4 s each, with an overlap of 2 s. The geometry consists of 2048 notes, each of which may or may not host HDF, and may or may not host HRI. Across the 13 time windows, there are therefore 2048 * 13 = 26624 opportunities for HDF + HRI concurrence per patient. A period of HDF + HRI concurrence is considered as a spatially and temporally contiguous period of HDF + HRI concurrence of at least 1 time window, at any single node. With this in mind, the median (range) of HDF + HRI concurrence periods was 128.5 (0–628) out of a possible 26624 occurrences, per patient.

When considering all patients together, in this study there were a total of 1952 periods of HDF + HRI concurrence. The median duration of HDF + HRI concurrence was 1 time window (of 4 s), range 1–3 windows, i.e., 4–8 s (after accounting for window overlap). Importantly, only 82 out of the 1952 periods (4.2%) of HDF + HRI concurrence lasted for more than 1 time window.

### Analysis of Isopotential Maps

The numbers of activation events per patient across all 51 ablation regions observed on 30 s pre-ablation isopotential maps are summarized in Table 3.

**TABLE 3:** Frequency of left atrial activation events during lesion-by-lesion visual assessment of isopotential maps within each patient.

Patient	1	2	3	4	5	6	7	8	9	10	Total
Rotor	0	0	0	0	0	4	0	0	0	0	4
Critical pathway	21	0	12	6	7	127	11	3	86	42	315
Wavelet propagation	1	0	0	2	0	10	0	0	5	2	20
Focal activation	23	1	37	60	70	70	27	25	167	57	537
Focal extinction	27	1	18	48	59	41	27	19	121	16	377

*See text for definitions of activation behavior.*

**FIGURE 6:**
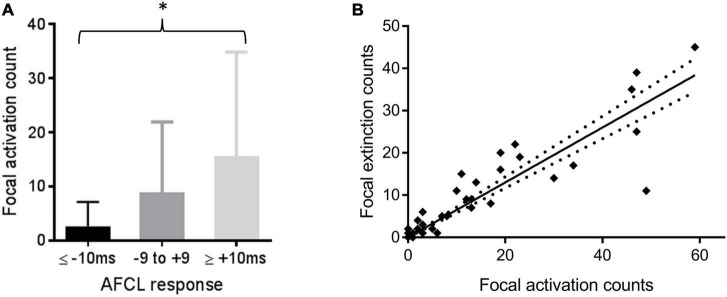
**(A)** Comparing numbers (mean and SD) of focal activation events with the AFCL response to ablation of that region; *p < 0.05 for Kendall’s correlation between the variables. **(B)** Counts of focal extinction and activation events within the same regions. Line of best fit and confidence intervals by linear regression are shown (*p* < 0.0001).

A positive association between the proportion of HDF occurrences and all isopotential events within ablated regions was mainly driven by the focal activation group (τ = 0.40, *p* < 0.0001). Focal event rates were indicatively different between ablation response groups (Figure 6A), and their ablation was weakly associated with an organizing AFCL response (τ = 0.21, *p* = 0.04).

Focal extinction events were strongly correlated with focal activations in the same region (τ = 0.79, *p* < 0.0001, Figure 6B). 0.65 extinction events (95% confidence intervals 0.58–0.71, *p* < 0.0001) were estimated to occur for every activation event in the same region. Rotor behavior was only observed 4 times during this study, and only in one patient (Patient 6). 3 of these 4 rotors occurred in the same ablated region. This particular region also recorded the highest overall number of wavefront activation events (excluding extinction events) in the whole study.

**FIGURE 7:**
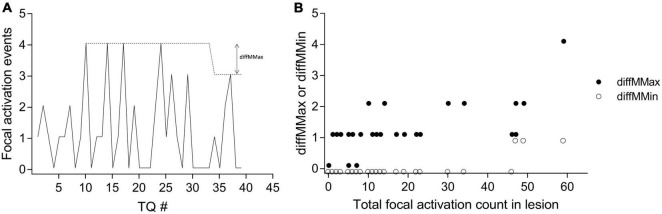
Demonstrating the temporal consistency of focal activation events. **(A)** In one patient, for each consecutive TQ interval, the number of observed focal activation events for one ablated region is shown by the solid line. The dotted line indicates the MMax (moving maximum) for the region, and an example of the derivation of diffMMax is shown. **(B)** The variation in the consistency of focal activation as measured by diffMMax (filled circles) and diffMMin (unfilled circles) across all 51 lesions from all 10 patients are shown. See main text for definitions.

The maximum and minimum number of focal activation events occurring in any given ablation region appeared to be consistent over time (example in Figure 7A). diffMMin values ranged from 0 to 1 only, whilst all diffMMax values were 2 or less, except one. Greater variability (i.e., higher diffMMax and diffMMin) tended to occur only with higher mean event rates. (τ = 0.73 and 0.41, respectively, *p* < 0.0001 for both, Figure 7B).

## Discussion

The present study shows that spatiotemporally dynamic HDF areas throughout the LA during human *in vivo* persAF can be prospectively, feasibly, and efficaciously targeted using a global multisite mapping approach based on an established commercial platform, even before PVI. 39% of HDF-targeted lesions resulted in an AFCL increase of 10 ms or more. The presence of focal activations on isopotential mapping was the most commonly observed electrophysiological behavior, and co-localized with HDF activity during AF in ablation regions. These activations were consistently observed in the same areas. Focal extinction events were strongly associated with focal activation events in these same areas, while rotor events were rare.

Regions with lower HRI occurrences were associated with a negative AFCL response to ablation, but HDF occurrences were not predictive. Simultaneous concurrence of HDF and HRI in the same time window and spatial location was uncommon and short-lived.

### Dynamic Highest Dominant Frequency Mapping Does Not Identify Clinically Relevant Rotor Behavior

Dominant frequency is implicated across multiple potential mechanisms of AF persistence including multiple loop re-entry (Lin et al., 2005), focal sources (Takashima et al., 2010) and rotors (Mandapati et al., 2000; Mansour et al., 2001), yet previous results from DF-targeted persAF ablation have been disappointing (Atienza et al., 2009, 2014; Verma et al., 2011). Part of the explanation lies in the temporal-spatial variability in DF (Yokoyama et al., 2009; Habel et al., 2010; Jarman et al., 2012; Salinet et al., 2014) which may have limited the point-by-point approaches that have been employed in many studies to date, and underpinned our belief that a panoramic whole-atrial method would be necessary for robust spectral mapping of persAF. However, despite using such an approach, prospective ablation of dynamic HDF targets in the present study did not predict AF organization.

While previous retrospective data alluded to this possibility (Jarman et al., 2012) the current study is the first to prospectively reach this conclusion. Early data from the cholinergic stimulation of sheep atria (Kalifa et al., 2006; Filgueiras-Rama et al., 2012) first proposed the relevance of micro-reentrant phenomena producing spatial frequency gradients which might be potentially mapped in the frequency domain. Subsequent evidence supported the concept of such “rotor” meandering around anatomical or recurrent functional areas of block (Gianni et al., 2016; Salinet et al., 2017), or varying in response to the autonomic milieu (Atienza et al., 2006), both of which would lead to dynamic DF behavior and hence require similarly dynamic mapping to target successfully.

It was hypothesized that the present study might clarify this through the combination of isopotential activation map analysis alongside HDF. However, during the comprehensive isopotential map analysis of ablated regions in the present work, only 4 rotor-like events were observed, all in the same patient. This is comparable to the published rates of similarly described behavior using the same technology (Yamabe et al., 2016). Our observation suggests that where rotors do arise, they may co-localize with (and could thus confound the targeting of) other activation phenomena. Overall though, the rarity of this type of rotor behavior, coupled to the overall equivocal AFCL outcomes with prospective dynamic HDF targeting, questions the significance of such phenomena in relation to both HDF mapping and human AF persistence, as detected using the current study platform. Direct rotor observation and ablation in humans (Narayan et al., 2012, 2014) has been controversial (Benharash et al., 2015; Gianni et al., 2016) and some groups using direct atrial patch electrodes during cardiac surgery have not observed rotor phenomena at all (Moe et al., 1964; Allessie et al., 2010; Lee et al., 2015).

In addition, the definition of a rotor is still debated. A popular approach is to generate instantaneous phase signals from time series data using the Hilbert transform (Umapathy et al., 2010). To “unmask” the rotational behaviors within narrower frequency ranges, pre-processing methods have been applied to intracardiac data before Hilbert transform. Wavelet/sinusoidal reconstruction and band-pass filters centered on DFs are examples of techniques for filtering out undesirable and/or non-physiologic activations (Rodrigo et al., 2014; Kuklik et al., 2015). Once robust phase mapping has been obtained, another factor to consider is the definition of a rotor in terms of completeness of rotations. While the original idea is of a re-entrant circuit requiring a full rotation with 1 cycle or 360 degrees, in practice, this is usually not achievable due to spatial electrodes sampling. More recently, a rotor with >75% of a full rotation was considered to be generally acceptable (Kowalewski et al., 2018). In the present study, the rotors were defined by visual assessment of isopotential maps in a manner similar to that of Yamabe et al. (2016). It is nevertheless possible that we could have underestimated the number of rotors that were present, as using activation or isopotential maps alone, based on electrograms or activation wavefronts, may have a tendency to overlook phase-singularity events that have been used to define rotors (Narayan and Zaman, 2016).

### Identifying Spectral Organization May Minimize Excess Ablation

The data in the present study shows that HDF-guided ablation may not always result in AF organization; in another words, HDF-guided mapping results in false-positive substrate identification. Interestingly however, where HDF-guided ablation resulted in AF disorganization, the pre-ablation HRI in these areas was significantly lower than if AF had organized, and to a lesser extent than if there was no AFCL response. Therefore, low HRI may have utility as an adjunctive indicator to avoid the risks of ineffective ablation of false-positive targets identified by HDF, or indeed by other putative substrate markers.

Dominant frequency variability is known to be inversely associated with spectral measures of AF organization (Takahashi et al., 2006; Jarman et al., 2014; Honarbakhsh et al., 2018b). As such, atrial zones with low HRI may be expected to host substantially more DF variation, which would not be consistent with putative source-like behavior. The fact that HDF and HRI were only very rarely spatiotemporally coincident in our cohort thus further supports a significantly lesser role for HDF than was previously assumed.

Relatively few studies have specifically evaluated the spectral assessment of organization in the context of AF ablation. Computer simulation has suggested that OI (organization index, a measure of spectral organization similar to the RI used in the present study) would be superior to DF in localizing focal activity (Everett et al., 2001; Tobon et al., 2012), Tuan et al. (2010) noted a rise in OI prior to AF termination with flecainide, with Takahashi and colleagues observing the same after isolation of a driving PV in pAF (Takahashi et al., 2006). Jarman and colleagues documented in 6 patients, also using a non-contact array in the LA, that where PVI with wide area circumferential ablation had coincidentally crossed areas of higher organization, the organization in a distal part of the LA (around the LAA) also increased (Jarman et al., 2014). However, the organization in adjacent sites did not change significantly which may run counter to the idea of the index area as an AF source.

More recently, Honarbakhsh et al. (2019) used a 64-pole basket contact catheter and CARTOFINDER to evaluate 44 AF driver sites in 29 patients, defined by either rotational or focal activity observed over 30 s. Following PVI, 39 out of 44 prospectively ablated driver sites resulted in AFCL prolongation (of at least 30 ms) or termination. Interestingly the sensitivity (true positive rates) for HDF and HRI were 50 and 95%, while false positive rates were 37 and 33%, respectively.

### Epicardial-Endocardial Interaction: An Alternative Hypothesis for Highest Dominant Frequency in Atrial Fibrillation Persistence

Our method of tracking HDF did not assume any specific underlying electrophysiological mechanism other than the relevance of high frequency activation sites in maintaining persAF. To explore this further, we investigated the underlying isopotential patterns within ablated regions, seeking pre-defined mechanistic behaviors that co-localized with or formed the basis for HDF events or for the AFCL response to ablation.

Out of all our pre-defined activation patterns, only focal activation events were found to be associated with AFCL response, and more interestingly also (very strongly) with focal extinction events. The co-localization of focal activation and extinction suggests that the same anatomical regions may act as both source and sink in the electrophysiological environment, where current can both originate from and flow back to. Our results suggest the possibility of other electrophysiologically active tissue permitting the channeling of current both toward and away from the endocardium – in other words, multiple electrophysiologically relevant myocardial layers. To the best knowledge of the authors, this is the first presentation of data from a commercially available mapping system in the LA that is supportive of the multi-layer hypothesis in human persAF (de Groot et al., 2010, 2016). In keeping with this interpretation and their own conclusions, de Groot and colleagues (de Groot et al., 2016) documented highly correlated numbers of focal endocardial and epicardial events measured using contact electrodes in the right atrium during AF in cardiac surgery (R^2^ = 0.89, *p* < 0.0001, our calculation). Not all focal waves breaking through to the epicardium will originate from the endocardium, which may explain the apparent shortfall of endocardially observed extinction events compared to activation events in the present work. Notably, 57% of our ablated regions demonstrated repetition of focal behavior, often with clear anatomical consistency even within the ablated area (see example in Supplementary Video 4), whereas < 10% of focal events in the data from de Groot et al., were repetitive, probably due to differences in detection criteria, and a shorter mapping time of 10s per patient. Our data suggests that 30 s would be sufficient to observe temporally consistent focal activity in humans.

We also show for the first time an association between HDF events and observed focal events. Computer modeling studies (Gharaviri et al., 2017) suggest that reducing the number of epicardial-endocardial breakthrough sites (BTRs) could increase or decrease AF stability. Although this study could not look specifically at BTRs ablation, our finding of a heterogeneous AFCL response to ablation in potentially equivalent areas is supportive of this and may have contributed to the equivocal outcomes from previous DF-targeted persAF ablation studies.

### Limitations

We believe our work on a small number of patients offers a number of useful insights into persAF behavior in the context of HDF ablation, but larger patient cohorts would be needed to confirm or otherwise the prospective validity of future similar methodologies.

Isopotential map analysis was voltage thresholded at a level which may have precluded visualization of lower amplitude but electrophysiologically relevant signals. It is however notable that the correlation between focal activation and extinction events was preserved (τ = 0.82, *p* < 0.0001) even when the threshold for activation was reduced (i.e., made more stringent) from −0.28 to −0.53 mV, negating the idea of a noise-driven phenomenon, and suggesting that the −0.28 mV threshold was reasonably specific for the detection of this type of behavior.

An average of 10% of Array geometry points were located more than 40 mm from the Array, at which point signal quality is known to decrease (Kadish et al., 1999). The process of HDF evaluation will be partially resistant to this effect (Gojraty et al., 2009), as it is less dependent on signal amplitude.

Ablation can alter spectral characteristics at distant sites (Jarman et al., 2014; Salinet et al., 2017), therefore it is possible that the cumulative effect of sequentially targeted ablation may be different to each lesion considered individually. The effect of this was partially accounted for with analysis for 2,3 and all available lesions separately as shown in Figure 5. In the future, faster generation of global DF maps may increase the feasibility of applying an iterative approach (remapping after each lesion is delivered) to investigate this further.

The current investigation was focused on frequency domain analysis. Future work including other metrics such as entropy and coherence could bring new insights and help to better understand the underlying mechanisms of persAF (Lee et al., 2013; Almeida et al., 2017, 2018; Li et al., 2020, 2021).

In the absence of confirmatory epicardial data, the endo-epi interaction shown through non-contact mapping was observational in nature and hence hypothesis generating only. Computational simulation or pre-clinical experiments may provide more evidence but were not included in the current study.

## Conclusion

We have shown that the ablation of spatiotemporally dynamic HDF regions guided by global intra-cardiac non-contact mapping is feasible and can acutely organize persAF before PVI. However, HDF alone has inadequate specificity for AF driving sites. During persAF ablation, left atrial areas of low organization in the frequency domain are unlikely to be appropriate substrate targets and should be avoided to reduce excess ablation and its consequences. Whole-chamber non-contact mapping may be able to detect epicardial-endocardial interactions in persAF, but further studies are needed to better delineate the importance of this in clinical practice.

## Data Availability Statement

The raw data supporting the conclusions of this article will be made available by the authors, without undue reservation.

## Ethics Statement

The studies involving human participants were reviewed and approved by United Kingdom NHS National Research Ethics Service. The patients/participants provided their written informed consent to participate in this study.

## Author Contributions

GC: concept/design study, data analysis/interpretation of results, drafting manuscript, critical revision of manuscript, statistics, and “off-line” data collection. XL: concept/design study, data analysis/interpretation of results, drafting manuscript, critical revision of manuscript, and statistics. PS: EP studies and ablation procedures, concept/design study, EP study, data collection, interpretation of results, and critical revision of manuscript. FV: data analysis/interpretation of results, critical revision of manuscript, and statistics. JS, AS, and PK: data analysis/interpretation of results and critical revision of manuscript. TA: data analysis/interpretation of results, drafting manuscript, and critical revision of manuscript. ND: data analysis/interpretation of results, and critical revision of manuscript. FS: concept/design study, data analysis/interpretation of results, and critical revision of manuscript. GN: EP studies and ablation procedures, concept/design study, interpretation of results, and critical revision of manuscript. All authors contributed to the article and approved the submitted version.

## Conflict of Interest

The authors declare that the research was conducted in the absence of any commercial or financial relationships that could be construed as a potential conflict of interest.

## Publisher’s Note

All claims expressed in this article are solely those of the authors and do not necessarily represent those of their affiliated organizations, or those of the publisher, the editors and the reviewers. Any product that may be evaluated in this article, or claim that may be made by its manufacturer, is not guaranteed or endorsed by the publisher.
